# The Effect of Antigen Stimulation on the Migration of Mature T Cells from the Peripheral Lymphoid Tissues to the Thymus

**DOI:** 10.1155/2001/20728

**Published:** 2001

**Authors:** Charles L. Hardy, Dale I. Godfrey, Roland Scollay

**Affiliations:** 1 Centenary Institute of Cancer Medicine and Cell Biology Locked Bag no 6 Newtown NSW 2042 Australia; 2 Department of Pathology and Immunology Monash University Medical School Commercial Rd. Prahran VIC 3181 Australia; 3 SyStemix, Inc. 3155 Porter Drive Palo Alto CA 94304 USA

**Keywords:** Thymus, T lymphocytes, Homing, Recirculation, Transgenic

## Abstract

Although the maturation and export of T cells from the thymus has been extensively studied, the movement of cells in the opposite direction has been less well documented. In particular, the question of whether T cells which have been activated by antigen in the periphery are more likely to return to the thymus had been raised but not clearly answered. We examined this issue by activating T cells present in the periphery with their cognate antigen, and assessing migration to the thymus. TCR-transgenic cells from OT-I mice (Thy1.2^+^), which recognise the ovalbumin peptide OVA_257–264_ in the context of H-2K^b^, were transferred into otherwise unmanipulated Thy1.1^+^ C57BL/6 mice. Recipient mice were injected i.v. with 5 μg peptide (SIINFEKL) approximately 24 hours later. The numbers of donor-derived (Thy1.2^+^) cells in the thymus and peripheral lymphoid tissue were determined. The results clearly show increased numbers of transgenic cells in the thymus 3 days after antigenic stimulation. However, since numbers of transgenic cells increased in the spleen and LN in about the same proportion, the data do not support the notion that there is highly increased selective migration of activated T cells to the thymus. Rather, they suggest that a sample of peripheral cells enters the thymus each day, and that the mature immigrants detected in the thymus merely reflect the contents of the peripheral T cell pool.


*Developmental Immunology*, 2001, Vol. 8(2), pp. 123-131
Reprints available directly from the publisher
Photocopying permitted by license only

© 2001 OPA (Overseas Publishers Association)
N.V. Published by license under
the Harwood Academic Publishers imprint,
part of The Gordon and Breach Publishing Group,
a member of the Taylor and Francis Group

### The Effect of Antigen Stimulation on the Migration of Mature T Cells from the Peripheral Lymphoid Tissues to the Thymus*

CHARLES L. HARDY†, DALE I. GODFREY‡ and ROLAND SCOLLAY¶

*From the Centenary Institute of Cancer Medicine and Cell Biology, Locked Bag no 6, Newtown, NSW, Australia, 2042*

Although the maturation and export of T cells from the thymus has been extensively studied, the movement of cells in the opposite direction has been less well documented. In particular, the question of whether T cells which have been activated by antigen in the periphery are more likely to return to the thymus had been raised but not clearly answered. We examined this issue by activating T cells present in the periphery with their cognate antigen, and assessing migration to the thymus. TCR-transgenic cells from OT-I mice (Thy1.2$^+$), which recognise the ovalbumin peptide $OVA_{257-264}$ in the context of H-2K$^b$, were transferred into otherwise unmanipulated Thy1.1$^+$ C57BL/6 mice. Recipient mice were injected i.v. with 5 μg peptide (SIINFEKL) approximately 24 hours later. The numbers of donor-derived (Thy1.2$^+$) cells in the thymus and peripheral lymphoid tissue were determined. The results clearly show increased numbers of transgenic cells in the thymus 3 days after antigenic stimulation. However, since numbers of transgenic cells increased in the spleen and LN in about the same proportion, the data do not support the notion that there is highly increased selective migration of activated T cells to the thymus. Rather, they suggest that a sample of peripheral cells enters the thymus each day, and that the mature immigrants detected in the thymus merely reflect the contents of the peripheral T cell pool.

*Keywords:* Thymus, T lymphocytes, Homing, Recirculation, Transgenic

*Abbreviations:* CFSE, carboxy fluorescein (diacetate) succinimidyl ester; LN, lymph node; MLN, mesenteric lymph node; OT-I mice, $OVA_{257-264}$-specific TCR-transgenic mice; $OVA_{257-264}$, ovalbumin peptide

### INTRODUCTION

The thymus is the primary source of T cells for the peripheral lymphoid organs. T cells produced in the thymus migrate to the spleen and lymph nodes (LN), especially early in life, and in the absence of a thymus these organs remain essentially devoid of T cells. This pathway has been extensively quantitated in a number

* This work was supported by grants from the National Health and Medical Research Council of Australia.

† Corresponding author and current address: Department of Pathology and Immunology, Monash University Medical School, Commercial Rd., Prahran, VIC, Australia, 3181. Tel: +61 3 9903 0541. Fax: +61 3 99030731. Email: charles.hardy@med.monash.edu.au

‡ Current address: Department of Pathology and Immunology, Monash University Medical School, Commercial Rd., Prahran, Victoria, Australia, 3181.

¶ Current address: SyStemix, Inc., 3155 Porter Drive, Palo Alto, CA, USA, 94304.

of species, and in young adult animals represents an export rate of about 1% of thymocytes per day (Scollay et al., 1980; Scollay and Shortman, 1985).

The reverse pathway, of mature T cells migrating from the periphery back into the thymus is less often considered, although several studies have shown that this is not a major pathway in normal animals (Dumont et al., 1984; Michie et al., 1988; Hirokawa et al., 1989; Agus et al., 1991). Our own unpublished data suggest that about 1% of the mature phenotype cells in the thymus may be mature T cell immigrants (Coward and Scollay, in preparation). Some studies have suggested that this pathway might preferentially be used by activated T cells. An early report indicated that activated antigen-specific T cells migrated to the thymus, and persisted there, whereas migration of non-activated cells to the thymus was not detected (Naparstek et al., 1982). Similarly, Fink et al. (1984) demonstrated that peripheral T cells activated *in vivo* provide a greater contribution to the thymic memory CTL response than non-activated cells. In that study, it was not possible to determine the proportion of thymocytes which were donor derived. More recently, it was shown that blasts (generated in parent $\rightarrow F_1$ combinations) homed to the thymus, and represented approximately 0.4% of mature T thymocytes, although the rate of homing was greatly increased by irradiation of hosts (Agus et al., 1991). Others have shown that, compared to 'naïve' $CD4^+$ T cells, there is a preferential accumulation of antigen-experienced T cells in the rat thymus (Bell et al., 1995; Westerman et al., 1996). As is the case for 'normal' peripheral T cells (Hirokawa et al., 1989), accumulation of activated cells within the thymus is largely restricted to the medulla (Pabst and Binns, 1989; Agus et al., 1991; Westermann et al., 1996).

One shortcoming of the above studies is that thymic immigration of *in vivo*-activated clonal T cell populations has not been analysed. As part of a longer study on migration of mature cells into the thymus, we have addressed this question in a model where TCR transgenic T cells present only in the periphery could be stimulated with their cognate peptide, and their migration to the thymus assessed. OT-I mice (Hogquist et al., 1994) carry a transgene for a MHC class I-restricted TCR with Vα2 and Vβ5 variable regions. The TCR recognises the ovalbumin peptide $OVA_{257-264}$ presented by $H\text{-}2K^b$, the MHC haplotype of C57BL/6 mice. In these mice, the great majority of peripheral T cells are $CD8^+CD4^-$, and express the transgenic TCR (Koniaris et al., 1997). These cells are readily activated *in vivo* upon administration of the OVA peptide (Koniaris et al., 1997).

The data described in this paper clearly demonstrate increased numbers of transgenic cell in the thymus after peripheral antigenic stimulation, but since cell numbers are also increased in the periphery, the results do not support the concept that activated cells show selective migration to the thymus.

### RESULTS

### Phenotype of Donor Cell Inoculum

Phenotyping of pooled donor spleen and LN cells prior to transfer indicated that $CD8^+$ cells were far more prevalent than $CD4^+$ cells (CD4:CD8 ratio of approximately 1:9), and that the majority (97%) of $CD8^+$ cells expressed Vα2 chains in their TCR, and were therefore peptide specific (Figure 1A&B).

### Quantitation of Donor-Derived Cells in Peripheral Lymphoid Organs

Recipient animals were anaesthetised, and perfused with PBS and FCS. Spleen and Mesenteric LN (MLN) cell suspensions were prepared, and stained to quantitate and phenotype donor-derived cells. In control mice which had been injected with donor cells followed by PBS instead of peptide three days previously, a population of donor-derived cells could be clearly seen, which comprised approximately 2% of total splenocytes, or 10 to 21% of $CD8^+$ splenocytes (Figure 1C; Table I). Similarly, in the LN, donor-derived cells represented 3 to 5% of total LN cells, or 9 to 18% of $CD8^+$ cells (Figure 1E; Table I). Three days post-peptide injection, donor-derived cells represented 12 to 17% of total splenocytes, and

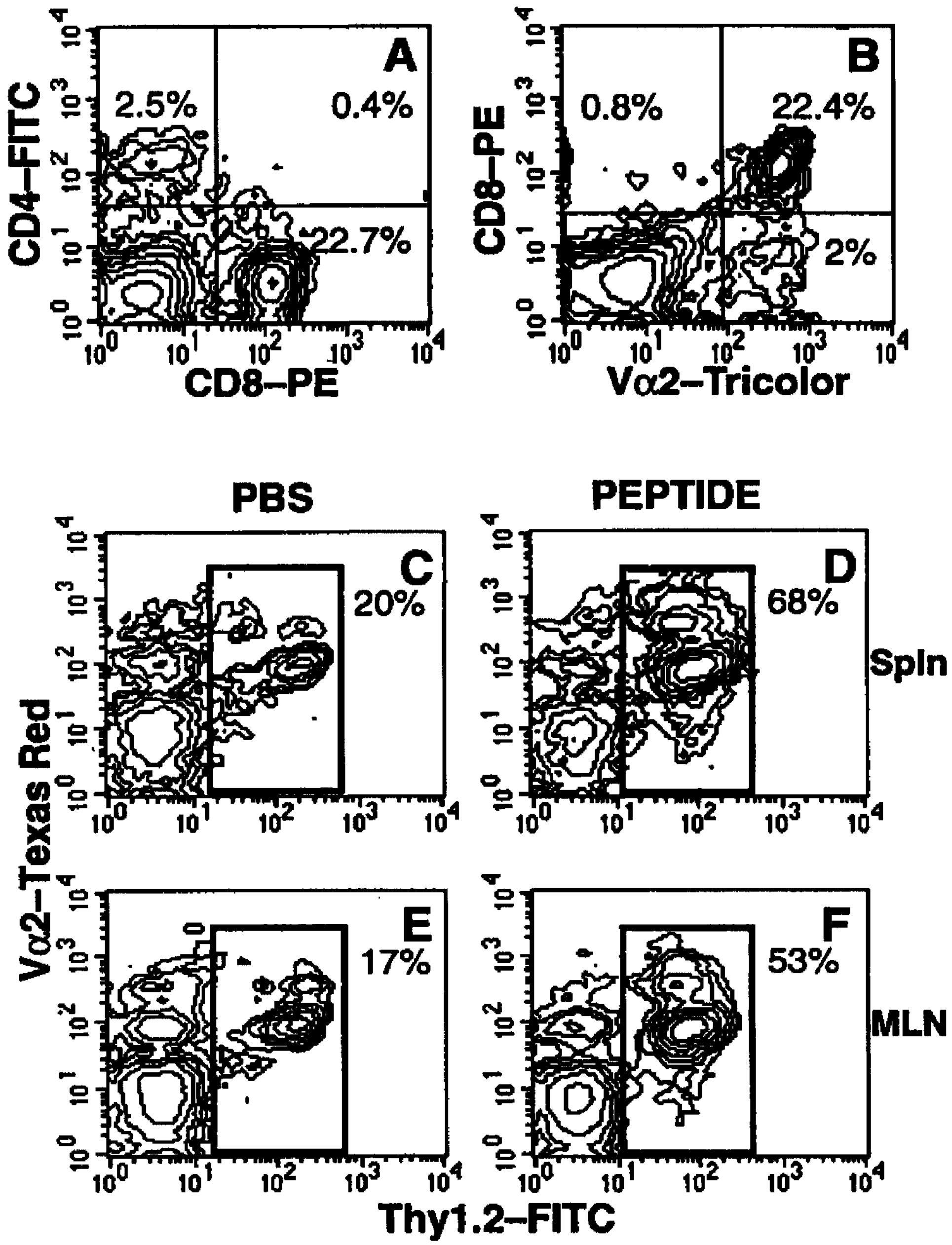

FIGURE 1 Phenotype of donor OT-I transgenic cells (A&B). Analysis for expression of donor marker (Thy1.2) and presence of transgenic TCR (Vα2) among gated CD8+ cells in recipient spleen and MLN 3 days after injection of PBS or peptide (C-F). (A) CD4 versus CD8 expression by donor lymphocytes prior to injection. (B) Vα2 expression by donor CD8 lymphocytes prior to injection. Representative of one donor cell inoculum, pooled from 13 OT-I donor mice. Plots from spleen of PBS (C) or peptide-injected (D) mice, and from MLN of PBS (E) or peptide-injected (F) mice. The proportion of total CD8+ lymphocytes that were donor-derived (Thy1.2+) is indicated. Representative of 5–6 mice approximately 66% of $CD8^+$ splenocytes. Three days post-peptide, donor-derived cells represented 27% of total LN cells, and approximately 50% of $CD8^+$ LN cells (Figure 1D&F; Table I). Thus, there was a large increase in donor-derived Ag-specific cells in peripheral lymphoid tissue three days after peptide injection. By day six after peptide, the proportion of total lymphocytes and $CD8^+$ lymphocytes which were donor-derived was similar to the control PBS-injected group (Table I).

When total cell numbers were considered, there was a 2 to 3-fold expansion in the number of splenocytes 3 days after peptide injection. This difference was not apparent 6 days after peptide, nor was it seen when LN cell numbers were compared. When absolute donor ($Thy1.2^+$) cell numbers were compared, there was a 15 to 20-fold expansion in spleens, and a 6 to 8-fold expansion in MLN 3 days after peptide injection, leading to an approximate 15 to 20-fold increase in the number of donor-derived cells in the total peripheral pool (based on the assumption that the total cell pool in all LNs is small relative to cell numbers in the enlarged spleen). Six days post-peptide there were negligible increases in absolute $Thy1.2^+$ cell numbers (Table I).

TABLE I Percentage and absolute numbers of splenocytes and LN cells which were donor-derived[a]

| *Group* | *% of total cells* | *% of $CD8^+$ cells* | *Total cell # ($10^6$)* | *$Thy1.2^+$ cell # ($10^6$)[b]* |
|---|---|---|---|---|
| **Experiment 1** | | | | |
| PBS spleen 3d | 2.2 +/- 0.1 (2.2, 2.3, 2.1)[d] | 10 +/- 1 (11, 9, 10) | 77 +/- 8 (84, 68, 78) | 1.7 +/- 0.1 (1.8, 1.6, 1.6) |
| Peptide spleen 3d | 11.6 +/- 1 (12.7, 11.6, 10.6) | 64 +/- 0.2 (64, 65, 64) | 228 +/- 61 (184, 298, 202) | 26 +/- 7.6 (23, 35, 21) |
| Fold increase | 5 x | 7 x | 3 x | 15 x |
| Control LN 3d | 2.9 +/- 0.2 (2.8, 3.1, 2.8) | 9 +/- 0.6 (9, 10, 9) | 17 +/- 4.9 (20, 11, 19) | 0.5 +/- 0.2 (0.6, 0.3, 0.5) |
| Peptide LN 3d | 25 +/- 3 (28, 22, 25) | 48 +/- 4.6 (53, 44, 47) | 15 +/- 5.9 (22, 13, 11) | 4 +/- 2 (6.3, 2.9, 2.9) |
| Fold increase | 9 x | 5 x | 0.9 x | 8 x |
| **Experiment 2** | | | | |
| Control spleen 3d | 1.3, 1.8[e] | 22, 20 | 114, 106 | 1.5, 1.9 |
| Peptide spleen 3d | 17.4 +/- 0.7 (17.6, 16.6, 18) | 67 +/- 3 (70, 64, 68) | 195 +/- 44 (152, 192, 240) | 34 +/- 8.4 (26.8, 31.9, 43.2) |
| Fold increase | 11 x | 3 x | 2 x | 20 x |
| Control LN 3d | 4, 5.7 | 17, 20 | 18, 19 | 0.7, 1.1 |
| Peptide LN 3d | 29 +/- 3.1 (28, 26, 32) | 53 +/- 3 (53, 50, 56) | 19 +/- 3 (18, 16, 22) | 5.5 +/- 1.4 (5.1, 4.3, 7) |
| Fold increase | 6 x | 3 x | 1 x | 6 x |
| Control spleen 6d | 1.2 +/- 0.3 (1.2, 0.9, 1.4)[e] | 12 +/- 0.6 (12, 11, 12) | 97 +/- 25 (72, 98, 122) | 1.2 +/- 0.5 (0.9, 0.9, 1.7) |
| Peptide spleen 6d | 1.6 +/- 0.8 (1.5, 2.5, 0.9) | 13 +/- 3 (14, 16, 10) | 121 +/- 15 (110, 138, 116) | 2.1 +/- 1.3 (1.7, 3.5, 1) |
| Fold increase | 1 x | 1 x | 1 x | 2 x |
| Control LN 6d | 2.9 +/- 0.3 (3.2, 2.6, 2.8) | 11 +/- 0.6 (11, 10, 11) | 13 +/- 3 (15, 15, 10) | 0.4 +/- 0.1 (0.5, 0.4, 0.3) |
| Peptide LN 6d | 2.3 +/- 1.0 (1.7, 3.4, 1.7) | 8 +/- 3.6 (5, 12, 7) | 20 +/- 6 (15, 27, 19) | 0.5 +/- 0.3 (0.3, 0.9, 0.3) |
| Fold increase | 0.8 x | 0.7 x | 1.5 x | 1 x |

a. Mean +/- one standard deviation (individual values).

b. Value derived from % total lymphocytes and cell number.

c. Student's t-test, control versus peptide-injected.

d. Values from spleen and MLN of control mice not infused with $Thy1.2^+$ cells (background with αThy1.2 Ab) were 0.05 - 1%.

e. Values from spleen and MLN of control mice not infused with $Thy1.2^+$ cells (background with αThy1.2 Ab) were 0.03 - 0.2%.

### Quantitation of Donor-Derived Cells in the Thymus

Following anesthesia, animals were perfused with PBS in order to flush out any circulating, donor-derived cells from the thymus. In control, PBS-injected mice (i.e. donor cell-injected but no peptide), 12 to 41 per $10^5$ thymocytes were donor-derived 3 days post-PBS injection, whilst 417 to 1043 per $10^5$ thymocytes were donor-derived (Thy1.2$^+$) in the peptide-injected group (Table II). These differences translated into 25 to 35-fold increases in the proportion of donor-derived cells in the peptide-injected group at the earlier time-point (Figure 2C&D; Table II). By day 6 after peptide, the difference in proportions of donor-derived cells was reduced to 3.5-fold (Figure 2E&F; Table II). In terms of total cell numbers, the thymuses of peptide-injected mice had slightly fewer cells than those of PBS-injected mice (Table II). However, compared to PBS-injected mice, the absolute number of donor-derived (Thy1.2$^+$) cells detected in the thymus was increased 16 to 20-fold 3 days after peptide administration (Table II). This difference was reduced to 3-fold at the latter time-point (Table II).

### Proportions of Transgene-Expressing (V$\alpha$2$^+$) Donor-Derived CD8$^+$ Cells

In order to ensure that donor-derived cells were indeed potentially antigen responsive, we assessed the proportion of donor CD8$^+$ cells which were V$\alpha$2$^+$. Apart from slightly lower values in the thymus at day 6 after peptide administration, essentially all (approximately 95%) CD8$^+$ cells expressed the transgenic V$\alpha$2 chain in their TCR (Table III), and were therefore antigen-specific.

TABLE II Proportional and absolute number of thymocytes which were donor-derived[a], three and six days after peptide stimulation

| *Group* | *Immigrants/$10^5$ cells*[b,c] | *Total cell # ($10^6$)* | *Thy1.2$^+$ cell # ($10^4$)*[d] |
|---|---|---|---|
| **Experiment 1** | | | |
| PBS | 12 +/- 4 (16, 13, 8) | 169 +/- 37.2 (204, 130, 174) | 2 +/- 1 (3, 2, 1) |
| Peptide | 417 +/- 139 (576, 358, 318) | 95 +/- 13.3 (104, 80, 102) | 40 +/- 17 (60, 29, 32) |
| Fold increase | 35 x | 0.6 x | 20 x |
| **Experiment 2** | | | |
| PBS 3d | 30, 52 | 176, 214 | 5, 11 |
| Peptide 3d | 1043 +/- 283 (1159, 720, 1249) | 119 +/- 38 (84, 114, 160) | 127 +/- 64 (97, 82, ,200) |
| Fold increase | 25 x | 0.6 x | 16 x |
| PBS 6d | 35 +/- 4 (30, 38, 37) | 133 +/- 36 (108, 174, 118) | 5 +/- 2 (3, 7, 4) |
| Peptide 6d | 121 +/- 13 (117, 135, 110) | 112 +/- 14 (128, 104, 104) | 13.5 +/- 2 (15, 14, 11) |
| Fold increase | 3.5 x | 0.8 x | 3 x |

a. Mean +/- one standard deviation (individual values).

b. Background values (no $\alpha$Thy1.2 Ab) were 0.1 - 1 per $10^5$ cells.

c. Values for control mice stained with $\alpha$Thy1.2 but not infused with Thy1.2$^+$ cells were 4 - 17 immigrants/$10^5$ cells.

d. Value derived from % immigrants and total cell number.

TABLE III Percentage of donor-derived (Thy1.2$^+$) CD8+ cells which were V$\alpha$2$^+$ [a]

| *Mouse (n)* | *Thymus* | *Spleen* | *MLN* |
|---|---|---|---|
| Control 3d (2) | 92.3 +/- 0.1 | 96.2 +/- 2.0 | 95.8 +/- 0.1 |
| Peptide 3d (3) | 97.9 +/- 0.2 | 94.9 +/- 0.2 | 95.4 +/- 0.6 |
| Control 6d (3) | 78.6 +/- 9.2 | 95.7 +/- 0.4 | 96.4 +/- 0.6 |
| Peptide 6d (3) | 97.2 +/- 1.8 | 95 +/- 1.2 | 96.4 +/- 0.7 |

a. Mean +/- one standard deviation.

### DISCUSSION

Although thymic maturation and rates of thymic export of mature T cells have been extensively studied (Scollay et al., 1980; Scollay and Shortman, 1985), the reverse pathway, of immigration of mature T cells into the thymus, has been less well analyzed. The current study aimed to determine whether T cells activated in the periphery show increased selective migration to the thymus. By activating peripheral T cells with specific antigen *in vivo* we could avoid some of the pitfalls of previous studies, in which activated cells were adoptively transferred (Naparstek et al., 1982; Fink et al., 1984; Agus et al., 1991; Bell et al., 1995; Westermann et al., 1996). While the cells involved had been transferred from congenic donors, and had suffered the rigors of being prepared as cell suspensions, they were not manipulated in any other way. This model is therefore relatively physiological, as there was no organ transplantation or recipient manipulation (apart from the i.v. injection), and provides a situation in which there is a population of T cells present in the periphery but absent from the thymus unless there has been immigration. Our data confirm experiments in other systems (Dumont et al., 1984; Michie et al., 1988; Hirokawa et al., 1989; Agus et al., 1991) which show that migration back to the thymus in normal, unstimulated animals is very low, and also confirm our own experiments using carboxy fluorescein (diacetate) succinimidyl ester (CFSE)-labeled cells from normal, non-transgenic donors which show the same thing (Coward and Scollay, in preparation). Those CFSE experiments also showed long-term survival and apparently normal migration of transferred spleen and LN cells in this experimental set up. Other studies show similar long-term survival and/or tissue-specific migration of fluorescein isothiocyanate-labeled cells (Butcher et al., 1980; Chin and Cahill, 1984; Abernethy et al., 1990; Witherden et al., 1990). For the experiments in this paper, the transferred T cells were allowed approximately 24 hours for 'recovery' before antigen stimulation in order to minimize any effects of the earlier *ex vivo* manipulation.

It was clear that the transferred transgenic T cells were stimulated to proliferate *in vivo* with the specific peptide, as evidenced by the loss of CFSE staining intensity (not shown) and the large increase in cell numbers. This was true three days after stimulation, although by 6 days, most of the donor cells had apparently disappeared, presumably due to antigen induced apoptosis, as shown in similar systems by several investigators (Webb et al., 1990; Kyburz et al., 1993; Moskophidis et al., 1993; Koniaris et al., 1997).

Three days after antigen administration, there were significant changes in the sizes of the lymphoid organs. The cellularity of the thymus was reduced, presumably due to the well characterised stress effects often associated with experimental manipulation, including delivery of antigen. The reduced thymic cellularity may have been due to deletion of cortical thymocytes, possibly mediated by cortico-steroids or TNF produced by peripherally activated T cells (Martin and Bevan, 1997). In contrast, the cellularity of the peripheral organs was increased, due to expansion of the transgenic T cells and cytokine-induced "bystander" proliferation (Tough et al., 1996; Ehl et al., 1997). For this reason we have focussed this discussion on absolute T cell numbers, as it is difficult to interpret the relevance of changes in proportion in the face of varying organ sizes.

There was clearly an increase in the number of cells which migrated into the thymus following antigen-specific T cell activation in the periphery, confirming the findings of earlier studies (Naparstek et al., 1982; Fink et al., 1984; Agus et al., 1991; Bell et al., 1995; Westermann et al., 1996). This increase amounted to about 16 to 20-fold 3 days after injection with peptide. However, at this same time point there had also been an increase in the number of cells in the periphery, by about 15 to 20-fold in the spleen and 6 to 8-fold in the MLN. In these animals with enlarged spleens, this must represent an increase in the total peripheral pool only slightly less than the 15 to 20-fold value seen in the spleen. Thus, the increase in the thymus (16 to 20-fold) was very close to that seen in the periphery (approximately 15 to 20-fold). Therefore, the results do not support the notion that there is preferential migration of activated peripheral T cells back to the thymus. Rather they support the idea that there may be a steady level of migration of activated

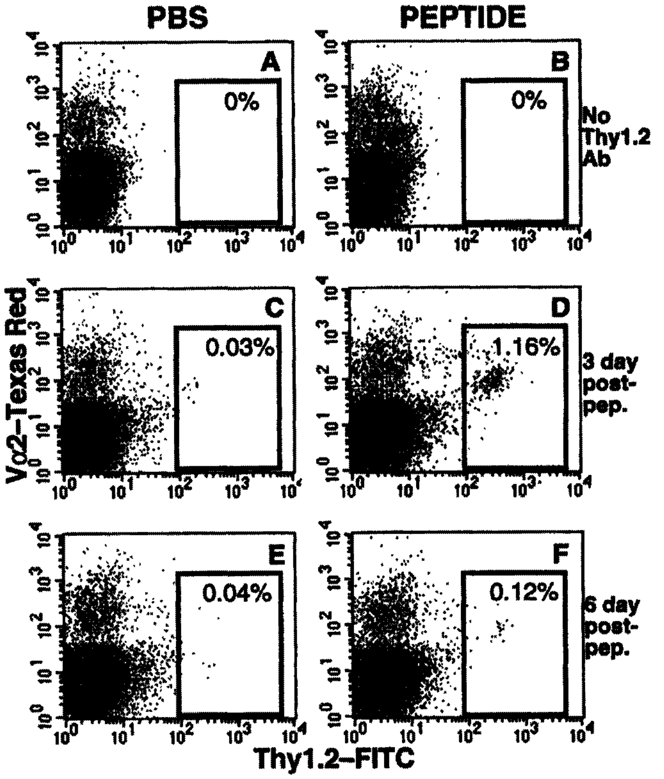

FIGURE 2 Identification of intrathymic, donor-derived (Thy1.2$^+$) transgene positive (Va2$^+$) cells in control and peptide-injected mice. Plots were generated by forward versus side light scatter gating on whole thymocytes. Background staining (no Thy1.2 Ab) 3 days after injection of PBS (A) or peptide (B). Plots from mice 3 days after injection of PBS (C) or peptide (D), or 6 days after injection of PBS (E) or peptide (F). Percentages of donor-derived cells are indicated. Representative of 3–6 mice peripheral cells to the thymus which is proportional to their numbers in the periphery. While it is clear that our data do not prove such a model, they are consistent with it, but not with a preferential migration model.

There is one additional point which should be made. We cannot exclude the possibility that the increased numbers of transgene-positive donor cells in the thymus result from proliferation inside this organ, rather than increased migration. Our data do not allow us to distinguish between these alternatives.

Previous investigations of immigration of mature T cells to the thymus were limited by the fact that activated cells were defined and purified on the basis of cell-surface marker expression (Bell et al., 1995; Westermann et al., 1996), or involved adoptive transfer of previously activated cells (Fink et al., 1984; Agus et al., 1991). Additionally, measurement of immigrating cells has frequently relied upon relatively non-quantitative radioactive, immunohistochemical or *in vitro* cell culture techniques (Naparstek et al., 1982; Fink et al., 1984; Westermann et al., 1996). By transferring large numbers of clonal T cell populations, administering specific peptide several days later, and quantitating thymic immigrants by flow cytometry we have been able to circumvent some of these shortcomings. Our results are in broad agreement with the previous studies, showing an enhanced accumulation of activated cells within the thymus, but for the first time allowing a comparison of changes in the pool from which the activated cells have come.

The functional significance of peripheral T cells in the thymus, especially previously activated T cells, is unknown. It has been suggested that, since the thymic medulla contains both epithelial and bone marrow-derived cells which express both MHC and co-stimulatory molecules (Picker and Siegelman, 1999), it is a potential site for immune reactions (Michie et al., 1988), although there is no clear evidence to support such a case. However, it is possible that the presence of activated cells in the thymus might play a role in intrathymic selection and maturation events, as suggested by several investigators (Naparstek et al., 1982; Michie et al., 1988; Agus et al., 1991). Such a mechanism would presumably require delivery to, and recognition of, peripherally-derived peptides within the medulla, which may selectively alter production of thymic emmigrants. The possibility that such peptide/self-antigen plays an important role in medullary proliferation or emigration remains to be demonstrated (see Scollay and Godfrey, 1995 for discussion).

### MATERIALS AND METHODS

### Mice

Normal female C57BL/6 Ka-Thy1.1 mice 8 wk of age were bred and housed under clean conditions in the Centenary Institute animal facility. Homozygous OT-I Vα2/Vβ5 TCR-transgenic mice, specific for $OVA_{257-264}$ (243–2 line, back-crossed to Thy1.2$^+$ C57BL/6 mice) (Hogquist et al., 1994) were the generous gift of Dr. Bill Heath (Walter and Eliza Hall Institute of Medical Research, Melbourne Australia). Donor OT-I mice were used at 2–4 months of age.

### Monoclonal Antibodies and Flow Cytometry

Approximately 3 × $10^6$ cells per sample were stained in 96 well U-bottomed tissue culture plates. Biotinylated Vα2 (clone B20.1) was obtained from Dr. Bill Heath. Thy1.2-FITC, CD4-APC, CD8-PE, αβTCR-PE, B220-PE and CD69-biotin (Pharmingen, San Diego, CA); streptavidin-Tricolour and CD4-Tricolour (Caltag Laboratories, Burlingame, CA), and streptavidin-Texas Red (Molecular Probes Inc., Eugene, OR) were purchased. Cells were labeled with appropriately diluted biotinylated primary antibody for 15–20 min, washed twice, and incubated with streptavidin-Tricolour and fluorescent-conjugated monoclonal antibodies for 15–20 min. Antibodies were diluted in 3% FCS in balanced salt solution (BSS). All staining volumes were 40 μl, and all steps were performed at 4°C. Flow cytometric data was acquired on a FACScan or FACStar (Becton Dickinson and Co., San Jose, CA).

### Cell Transfer and Peptide Injection

Spleen and LN were collected from one transgenic donor mouse per recipient, cell suspensions prepared, and red blood cells lysed for 5 min in ammonium chloride lysis solution. The cell suspension was pooled and washed in 3% FCS/BSS 3–4 times (400g for 7 minutes at 4°C), and viable cells counted in a hemocytometer. The cells (approximately $10^8$ from each donor) were resuspended in PBS and injected into the tail vein of recipient mice in a volume of 200 μl. Injection of donor OT-I cells was performed on 2 consecutive days, such that each recipient received a total of approximately 2 × $10^8$ cells. Recipient mice were injected intravenously (i.v.) with 5 μg peptide (SIINFEKL) in 200 μl PBS approximately 24 hours after the second transfer of OT-I cells.

### Harvesting of Tissues

Animals were anaesthetised with avertin (2% 2,2,2-Tribromoethanol and 2% *tert*-Amyl-alcohol [Aldrich Chemical Company, WI]), injected with 100 U of heparin, and perfused via the left ventricle with 25 ml of warmed (30–37°C) PBS containing 3% FCS and 5 U/ml heparin. This procedure removed most blood from the lung and thymus, leaving them a 'white' color, thus minimizing contamination of thymocytes with donor-derived cells present in the circulation. Other experiments (Coward and Scollay, in preparation) have demonstrated that blood contamination does not contribute significantly to thymocyte preparations made in this way. The thymus was harvested prior to spleen and MLN. Organs were passed gently through a stainless steel sieve with the end of syringe plunger, and washed twice in 3% FCS/PBS prior to counting. Care was taken at all stages to avoid contamination of thymus samples with cells from spleen or MLN.

### *Acknowledgements*

We thank Dr. Bill Heath at the Walter and Eliza Hall Institute of Medical Research (Melbourne, Australia) for generously providing the OT-I transgenic mice, and for helpful discussions.

### *References*

Abernethy, N. J., J. B. Hay, W. G. Kimpton, E. A. Washington and R. N. P. Cahill. 1990. Non-random recirculation of small, $CD4^+$ and $CD8^+$ T lymphocytes in sheep: evidence for lymphocyte subset-specific lymphocyte-endothelial cell recognition. *Int. Immunol.* 2:231.

Agus, D. B., C. D. Surh and J. Sprent. 1991. Reentry of T cells to the adult thymus is restricted to activated T cells. *J. Exp. Med.* 173:1039.

Bell, E. B., S. M. Sparshott and A. Ager. 1995. Migration pathways of CD4 T cell subsets *in vivo*: the $CD45RC^-$ subset enters the thymus via $\alpha_4$ integrin-VCAM-1 interaction. *Int. Immunol.* 7:1861.

Butcher, E. C., R. G. Scollay and I. L. Weismann. 1980: Direct fluorescent labeling of cells with fluorescein or rhodamine isothiocyanate. II. Potential application to studies of lymphocyte migration and maturation. *J. Immunol. Methods.* 37:109.

Chin, G. W. and R. N. P. Cahill. 1984. The appearance of fluorescein-labelled lymphocytes in lymph following *in vitro* or *in vivo* labelling: the route of lymphocyte recirculation through mesenteric lymph nodes. *Immunology* 52:341.

Dumont FJ. Barrois R. Jacobson EB. 1984. Migration of peripheral T and B cells into the thymus of aging (NZB × SJL)$F_1$ female mice. *Cell. Immunol.* 83:292.

Ehl, S., J. Hombach, P. Aichele, H. Hengartner and R. M. Zinkernagel 1997. Bystander activation of cytotoxic T cells: studies on the mechanism and evaluation of *in vivo* significance in a transgenic mouse model. *J. Exp. Med.* 185:1241.

Fink, P. J., M. J. Bevan and I. L. Weissman. 1984. Thymic cytotoxic T lymphocytes are primed *in vivo* to minor histocompatibility antigens. *J. Exp. Med.* 159:436.

Hirokawa K. Utsuyama M. Sado T. 1989. Immunohistological analysis of immigration of thymocyte-precursors into the thymus: evidence for immigration of peripheral T cells into the thymic medulla. *Cell. Immunol.* 119:160.

Hogquist K. A., S. C. Jameson, W. R. Heath, J. L. Howard, M. J. Bevan and F. R. Carbone. 1994. T cell receptor antagonist peptides induce positive selection. *Cell* 76:17.

Koniaris, C., S. R. M. Bennett, F. R. Carbone, W. R. Heath and A. M. Lew. 1997. Peptide-induced deletion of CD8 T cells *in vivo* occurs via apoptosis *in situ. Int. Immunol.* 9:1601–5.

Kyburz, D., P. Aichele, D. E. Speiser, H. Hengartner, R. M. Zinkernagel and H. Pircher. 1993. T cell immunity after a viral infection versus T cell tolerance induced by soluble viral peptides. *Eur. J. Immunol.* 23: 1956.

Martin, S. and M. J. Bevan. 1997: Antigen-specific and nonspecific deletion of immature cortical thymocytes caused by antigen injection. *Eur. J. Immunol.* 27: 2726.

Michie, S. A., E. A. Kirkpatrick and R. V. Rouse. 1988. Rare peripheral T cells migrate to and persist in normal mouse thymus. *J. Exp. Med.* 168:1929.

Moskophidis, D., F. Lechner, H. Pircher and R. M. Zinkernagel. 1993. Virus persistence in acutely infected immunocompetent mice by exhaustion of antiviral cytotoxic effector T cells. *Nature* 362: 758.

Naparstek, Y., J. Holoshitz, S. Eisenstein, T. Reshef, S. Rappaport, J. Chemke, A. Ben-Nun and I. R. Cohen. 1982. Effector T lymphocyte line cells migrate to the thymus and persist there. *Nature* 300:262.

Pabst R. and R.M. Binns. 1989. Heterogeneity of lymphocyte homing physiology: several mechanisms operate in the control of migration to lymphoid and non-lymphoid organs *in vivo. Immunol. Rev.* 108:83.

Picker, L. J. and M. H Siegelman. 1999: Lymphoid tissues and organs. In *Fundamental Immunology*, Fourth Edition. Paul, W. E. ed. Lippincott-Raven Publishers, PA, pp 486–494.

Scollay, R. and D. I. Godfrey. 1995. Thymic emigration: conveyor belts or lucky dips? *Immunol. Today*16:268.

Scollay, R. and K. Shortman. 1985. Cell traffic in the adult thymus: cell entry and exit, cell birth and death. In *Recognition and Regulation in Cell Mediated Immunity*. J. Watson and J. Marbrook, eds. Marcel Dekker, NY, pp. 31–60.

Scollay, R., E. C. Butcher and I. L. Weismann. 1980. Thymus cell migration. Quantitative aspects of cellular traffic from the thymus to the periphery in mice. *Eur. J. Immunol.* 10:210.

Tough, D. F, P. Borrow and J. Sprent. 1996. Induction of bystander T cell proliferation by viruses and type I interferon *in vivo. Science* 272:1947.

Webb, S., C. Morris and J. Sprent. 1990. Extrathymic tolerance of mature T cells: clonal elimination as a consequence of immunity. *Cell* 63:1249.

Westermann, J., T. Smith, U. Peters, T. Tschernig, R. Pabst, G. Steinhoff, S. M. Sparshott and E. B. Bell. 1996. Both activated and nonactivated leukocytes from the periphery continuously enter the thymic medulla of adult rats: phenotypes, sources and magnitude of traffic. *Eur J. Immunol.* 26:1866.

Witherden, D. A., W. G. Kimpton, E. A. Washington and R. N. P. Cahill. 1990. Non-random migration of $CD4^+$, $CD8^+$ and $\gamma\delta^+T19^+$ lymphocytes through peripheral lymph nodes. *Immunology* 70:235.

